# A Preclinical Alcohol Biobank: Samples from Behaviorally Characterized HS Rats for AUD Research

**DOI:** 10.1523/ENEURO.0207-25.2025

**Published:** 2025-09-09

**Authors:** Michelle R. Doyle, Paola Campo, Selen Dirik, Maria G. Balaguer, Angelica R. Martinez, Marsida Kallupi, Abraham A. Palmer, Giordano de Guglielmo

**Affiliations:** ^1^Department of Psychiatry, University of California San Diego, School of Medicine, La Jolla, California 92093; ^2^Institute for Genomic Medicine, University of California San Diego, School of Medicine, La Jolla, California 92093

## Abstract

Alcohol use disorder (AUD) imposes a significant global health burden, yet effective treatments remain limited. There are no well-characterized, AUD-relevant, rodent biological sample repositories to support research in this area. To address this gap, we established the Alcohol Biobank, a comprehensive resource containing thousands of samples from over 700 (half males, half females) genetically diverse heterogeneous stock (HS) rats. Modeled after two successful cocaine and oxycodone biobanks, this repository uses the chronic intermittent ethanol vapor exposure (CIE) model, paired with oral self-administration, to characterize AUD-like behaviors, including ethanol consumption, preference, motivation, and withdrawal symptoms such as allodynia and anxiety-like behavior. Longitudinal samples (blood, urine, and feces) are collected before, during, and after ethanol exposure, while tissue samples (brain, heart, kidneys, liver, cecum, reproductive organs, adrenal glands, blood) are obtained at intoxication, acute withdrawal, protracted abstinence, or from naive controls. Samples are preserved via snap-freezing or paraformaldehyde fixation to support diverse applications, including genomics, transcriptomics, proteomics, and neuroanatomy. Samples are freely available to nonprofit organizations at www.alcoholbiobank.org. Genetic and behavioral data about these rats are deposited in public repositories. The Alcohol Biobank facilitates collaborative research to uncover biomarkers and develop novel therapies for AUD, addressing a critical need in addiction science.

## Significance Statement

The Alcohol Biobank offers unprecedented access to biological samples from a genetically diverse rat population that have been thoroughly characterized on several behaviors related to alcohol use disorder (AUD). We are making these samples available free of charge to researchers who may not otherwise be able to perform these in-depth behavioral experiments but can use other techniques to understand the mechanisms underlying AUD with the goal of developing novel, effective therapeutics to advance our management and treatment of AUD.

## Introduction

Alcohol use disorders (AUDs) are pervasive in society, affecting an estimated 400 million people worldwide and contributing to ∼2.6 million deaths annually (World Health Organization, 2024). In the United States, ∼29.5 million individuals aged 12 and older suffer from AUD, yet only ∼7.6% receive any form of treatment (SAMHSA CfBHSaQC, 2023). The three FDA-approved treatments for AUD (disulfiram, naltrexone, and acamprosate) have modest efficacy and side effects that often limit use (Han et al., 2021). To improve current treatments and discover new ones, the individual differences in both the propensity to develop AUD-like behaviors and the response to treatment need to be better understood. A critical hurdle in identifying biomarkers and therapeutic targets is the limited number of repositories that include longitudinal samples from rodents subjected to sophisticated behavioral models of AUD.

To address this gap, we have created the Alcohol Biobank (www.alcoholbiobank.org) which contains thousands of samples from over 700 individual heterogeneous stock (HS) rats (Hansen and Spuhler, 1984) and will continue to grow. The Alcohol Biobank, modeled after the successful cocaine and oxycodone biobanks (Carrette et al., 2021), provides samples that can be used to identify biomarkers for AUD and facilitate the development of novel therapies by leveraging the genetic diversity and detailed behavioral characterization of these rats. While preclinical models have identified potential biomarkers for AUD, translating these findings to humans has been challenging due to small sample sizes, lack of standardized protocols across laboratories, and the use of inbred strains that fail to capture human genetic diversity. Each of the over 700 rats in the Alcohol Biobank is fully characterized for AUD-like behaviors in a controlled environment using state-of-the-art oral self-administration models paired with chronic intermittent ethanol vapor exposure (CIE), a well-established model of ethanol dependence (Roberts et al., 2000; Gilpin et al., 2008; Vendruscolo and Roberts, 2014; de Guglielmo et al., 2016, 2019, 2023; Doyle et al., 2024). Behavioral assessments include ethanol consumption, preference, motivation via progressive ratio schedules, and withdrawal symptoms like mechanical allodynia, anxiety-like behavior, and ethanol tolerance. These behaviors align with 8 of 11 DSM-5 criteria for AUD, enhancing the translational value of the findings.

The HS rats (Hansen and Spuhler, 1984) are also being used to perform genome-wide association studies (GWAS) to identify genetic variants associated with AUD-related traits, supported by whole-genome sequencing of each individual (Chitre et al., 2020; Gunturkun et al., 2022; Lara et al., 2024; King et al., 2025; Kuhn et al., 2025). Longitudinal samples, including blood, urine, and feces, are collected throughout the behavioral experiments. Terminal samples, such as brain, heart, kidneys, liver, cecum, reproductive organs, adrenal glands, and blood, are obtained at three key stages: during intoxication (13–15 h of ethanol vapor exposure), during acute withdrawal (7–10 h post-vapor), and after protracted abstinence (4 weeks post-vapor), as well as from naive rats. Samples are preserved using snap-freezing or paraformaldehyde fixation to ensure compatibility with various downstream applications.

The Alcohol Biobank is designed with a FAIR mindset (creative commons BY-NC), making samples freely available to nonprofit organizations at www.alcoholbiobank.org (Wilkinson et al., 2016). Complementary genetic and behavioral data about the same rats will be deposited in public repositories such as ratgenes.org, the Rat Genome Database (Smith et al., 2020) and Gene Network (Sloan et al., 2016). This resource is particularly valuable for researchers outside the addiction field who lack the infrastructure for complex behavioral studies, enabling them to access well-characterized samples for multiomics analyses and therapeutic testing.

Here, we introduce the Alcohol Biobank, providing methodological details and highlighting its potential applications to advance AUD research.

## Materials and Methods

### Subjects

Heterogeneous stock (HS) rats were sourced from Leah Solberg Woods (Wake Forest University; NMcwiWFsm #13673907, RRID:RGD_13673907) and Abraham Palmer (UC San Diego; McwiWfsmAap:HS #155269102, RRID:RGD_155269102). HS rats, developed at NIH in the 1980s by interbreeding eight inbred strains (Hansen and Spuhler, 1984) are maintained with >60 breeder pairs using a breeding scheme to minimize inbreeding (Solberg Woods and Palmer, 2019). Only one male and one female offspring per breeder pair were used for behavioral studies. At weaning, rats received radiofrequency identification (RFID) chips for individual tracking, scanned before experiments to ensure accurate data linkage. Rats were pair-housed under a 12 h reverse light/dark cycle (lights on at 20:00 non-dependent, 21:00 dependent phase) in temperature (20–22°C) and humidity (45–55%) controlled rooms. They had *ad libitum* access to tap water and chow (Envigo Teklad Rat Food Diet 8604) outside experimental sessions. Experiments, except training, occurred between 10:00 and 13:00 (1–5 h into dark cycle). Rats were handled at least three times before experiments.

### Drugs

A 10% v/v ethanol solution was prepared by mixing 95% ethanol with tap water, delivered at 0.1 ml/reinforcer. Quinine solutions were made by dissolving 0.1 or 0.3 g quinine hydrochloride dihydrate (Sigma-Aldrich) in 1 L of 10% v/v ethanol solution. For the loss of righting reflex task, a 20% v/v ethanol solution was created by mixing 95% ethanol with sterile saline and administered intraperitoneally at 2.5 g ethanol/kg body weight.

### Mechanical nociception

**Figure 1.**
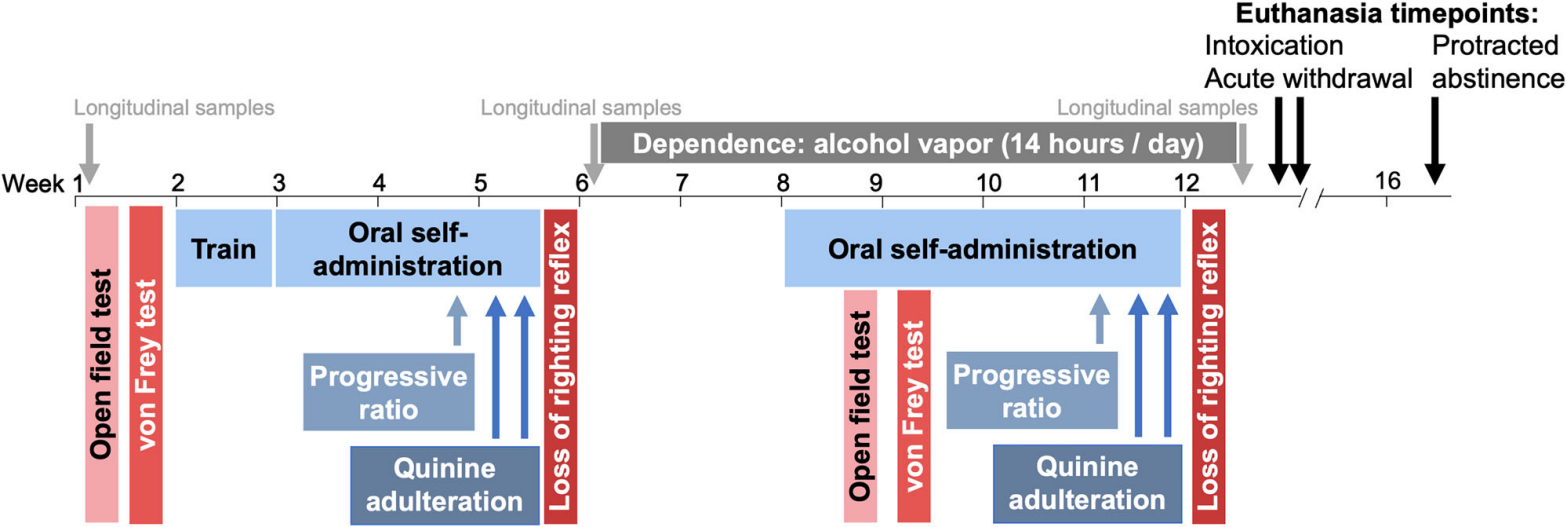
Experimental timeline. The time period where each behavioral assay is indicated. Alcohol consumption-related behaviors are in shades of blue and withdrawal-related behaviors are in shades of red. Longitudinal sample collection time points are indicated (before any alcohol exposure, after non-dependent drinking, and during acute withdrawal in dependent rats). Rats were euthanized at the conclusion of the study, during one of three time points.

Paw withdrawal thresholds were assessed at baseline (pre-ethanol exposure) and during acute withdrawal from ethanol dependence (Fig. 1). Rats habituated to the testing room for ≥30 min and to a clear chamber on a metal grid for ≥10 min. Mechanical stimulation used a dynamic plantar aesthesiometer (electronic von Frey, Ugo Basile), with the filament applied to the hindpaw center, increasing force from 0 to 40 g over 20 s. Thresholds were measured in triplicate per hindpaw, with ≥1 min between measurements. Time and force of paw retraction were averaged across both paws and triplicates.

### Open field test

Entries made into the center of an open field were measured for each rat at baseline (before any ethanol exposure) and during acute withdrawal from ethanol dependence (Fig. 1). Rats were allowed to habituate to the room for at least 30 min before testing began. The luminosity (lux) for the room lighting at time of test was measured through a Digital Lux Meter (Dr. Meter, model LX1330B) and set at 20 lux. Rats were placed into an open field [50 cm × 50 cm × 40 cm (height)] with black walls and gray floor and the center was defined as 16 cm × 16 cm area in the center of the open field. Behavior was tracked for 15 min using ANY-maze software.

### Loss of righting reflex (LORR)

Loss of righting reflex (LORR) duration was measured after a 2.5 g/kg ethanol injection (i.p.) before dependence induction (post-pre-dependent self-administration) and after dependent self-administration (Fig. 1). Time points recorded included injection, loss of righting reflex (rat placed on back), and reflex recovery (righting itself three times within 15 s). Baseline LORR (pre-ethanol exposure) indicated alcohol sensitivity, while the change in LORR from baseline to acute withdrawal measured ethanol tolerance.

### Self-administration procedure

#### Apparatus

Self-administration occurred in operant conditioning chambers (Med Associates) with two retractable levers on the right wall and a sipper cup with two wells between them. Levers were inserted at session start and remained accessible throughout. Two automated syringe drivers with 30 ml syringes delivered ethanol and water solutions to the right and left wells, respectively.

#### Training

Rats were trained to self-administer oral solutions in operant chambers during a 16 h session, receiving tap water (0.1 ml/reinforcer) on a fixed ratio (FR) 1 schedule (Fig. 1). Two days later, a second 16 h session delivered 10% v/v ethanol in tap water (0.1 ml/reinforcer) on an FR1 schedule. Chow was available *ad libitum*, with water or ethanol accessible only via active lever responses. Responses during the 0.6 s timeout were recorded but had no consequence.

#### Ethanol–water choice pre-dependence

Post-training, rats self-administered 10% v/v ethanol (right lever) or water (left lever) on an FR1:FR1 schedule during daily 30 min sessions, with each response delivering 0.1 ml of solution (Fig. 1). After nine daily (Monday–Friday) sessions, a progressive ratio (PR) test was conducted in a 45 min session with a 15 min limited hold. The response requirement increased by 1 every other infusion for the first 20 infusions, then by 1 per infusion (1, 1, 2, 2, 3, 3, …, 10, 11, 12…; Doyle et al., 2024), independently for each lever. This was followed by an ethanol–water choice session, a “low quinine” session (10% ethanol with 0.1 g/L quinine vs water), another ethanol–water choice session, and a “high quinine” session (10% ethanol with 0.3 g/L quinine vs water). The non-dependent phase spanned 14 sessions.

#### Self-administration during ethanol dependence

Following physical dependence induced by chronic intermittent ethanol vapor exposure (CIE), rats resumed self-administration on Mondays, Wednesdays, and Fridays during acute withdrawal (7–10 h post-vapor; Fig. 1). Ethanol and water were self-administered as in the non-dependent phase, but 12 sessions were conducted before a progressive ratio (PR) test, followed by low (0.1 g/L) and high (0.3 g/L) quinine sessions, with ethanol–water choice sessions interspersed. The dependent phase spanned 17 sessions. No self-administration sessions were conducted during or after the 4 week protracted abstinence period for rats assigned to this group, ensuring samples reflect a state of prolonged abstinence without recent ethanol exposure.

#### Chronic intermittent ethanol (CIE) vapor exposure to induce dependence

Rats were made physically dependent using the CIE model (Vendruscolo and Roberts, 2014; Kononoff et al., 2018; de Guglielmo et al., 2023; Doyle et al., 2024; Fig. 1). Rats were pair-housed (two per cage) in ethanol vapor chambers (La Jolla Alcohol Research) throughout the entire dependence phase. Ethanol vapor was circulated for 14 h/day, with a 10 h off period each day, during which the rats remained in the chambers. Rats were only removed from the chambers for self-administration sessions, which occurred during acute withdrawal (7–10 h after vapor exposure). Ethanol vapor was slowly increased over the course of 2 weeks, and blood ethanol concentrations (BECs) were measured twice per week until they reached an average of ∼180 mg/dl, with most individual subjects between 150 and 225 mg/dl.

#### Blood ethanol concentrations

BECs were measured by collecting 0.1 ml of tail blood into a heparinized tube after pricking the tail vein with an 18 G needle. Blood was spun in a centrifuge at 850 × *g* for 13 min to separate plasma. Plasma was then analyzed using gas chromatography (de Guglielmo et al., 2023) or using an Analox AM1 Alcohol Analyser.

#### Euthanasia during intoxication, acute withdrawal, or protracted abstinence

Rats were euthanized at one of three time points post-behavioral testing: intoxication (13–15 h of ethanol vapor), acute withdrawal (7–10 h post-vapor), or protracted abstinence (4 weeks post-vapor; Fig. 1). All rats underwent self-administration sessions during the dependence phase post-CIE induction, as described above. For rats euthanized during protracted abstinence, no self-administration or ethanol exposure occurred during the 4 week abstinence period prior to euthanasia. For snap-frozen samples, rats were euthanized via CO_2_ exposure, decapitated, and brains were flash-frozen in a 2-methylbutane/dry ice slurry; other tissues were frozen on dry ice. For fixed samples, rats were euthanized with CO_2_ and perfused intracardially with 150 ml ice-cold saline and 350 ml 4% paraformaldehyde in PBS, pH 7.4. Fixed brains were post-fixed in 4% PFA in PBS at 4°C for 24–72 h and then cryoprotected in 30% sucrose with 0.1% sodium azide in PBS. Longitudinal and terminal samples were collected from all experimental and naive rats (Table 1, Fig. 1).

**Table 1.** List of samples that are collected longitudinally (left column) or only at the terminal time point (right column)

Longitudinal samples	Terminal samples
Whole blood	Brain
Plasma	Heart
Serum	Liver
Urine	Kidney
Feces	Adrenal glands
	Ovary or testes
	Cecum
	PMBCs

### Statistical analysis

**Figure 2.**
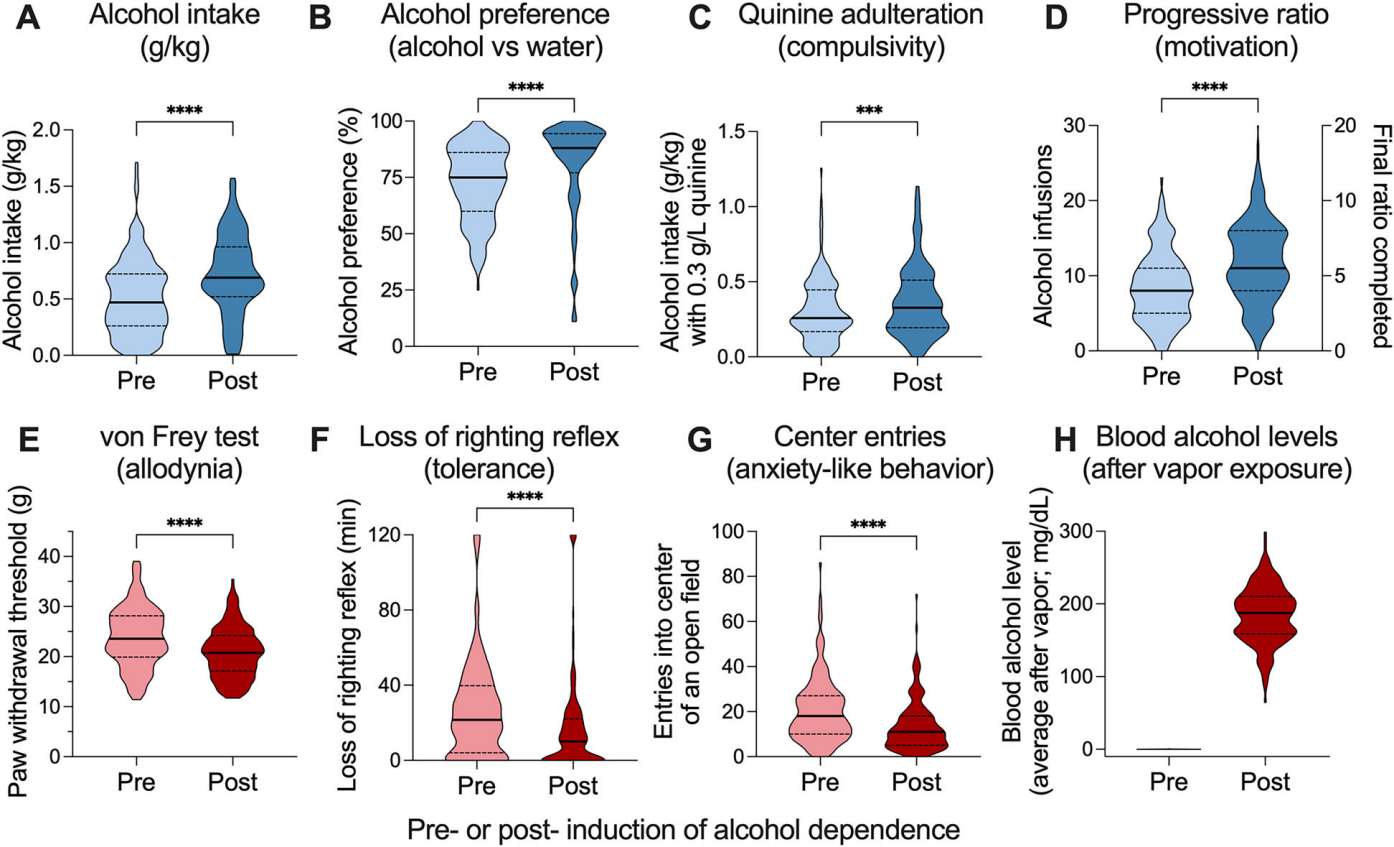
Comparisons between (***A***), alcohol intake (*n* = 190), (***B***), alcohol preference (*n* = 186), (***C***), alcohol intake when 0.3 g/L quinine was added to the alcohol (*n* = 190), and (***D***), motivation to obtain alcohol using the progressive ratio schedule of reinforcement in rats pre (light blue) or post (dark blue) induction of alcohol dependence (*n* = 190). Comparisons between (***E***) paw withdrawal threshold as a measure of withdrawal-induced allodynia (*n* = 190), (***F***) loss of righting reflex as a measure of tolerance (*n* = 177), and (***G***) center entries in an open field as a measure of withdrawal-induced anxiety-like behavior pre (light red) and post (dark red) induction of alcohol dependence (*n* = 183). ***H***, Blood alcohol levels at the end of the 14 h alcohol vapor exposure (*n* = 190). In violin plots, thick lines indicate median and thin lines indicate quartiles. Data represent combined results from two cohorts of 96 rats each (48 males and 48 females per cohort), totaling 190 rats after exclusions, with equal numbers of males (*n* = 95) and females (*n* = 95). ***p < 0.001; ****p < 0.0001 for paired *t* tests.

**Figure 3.**
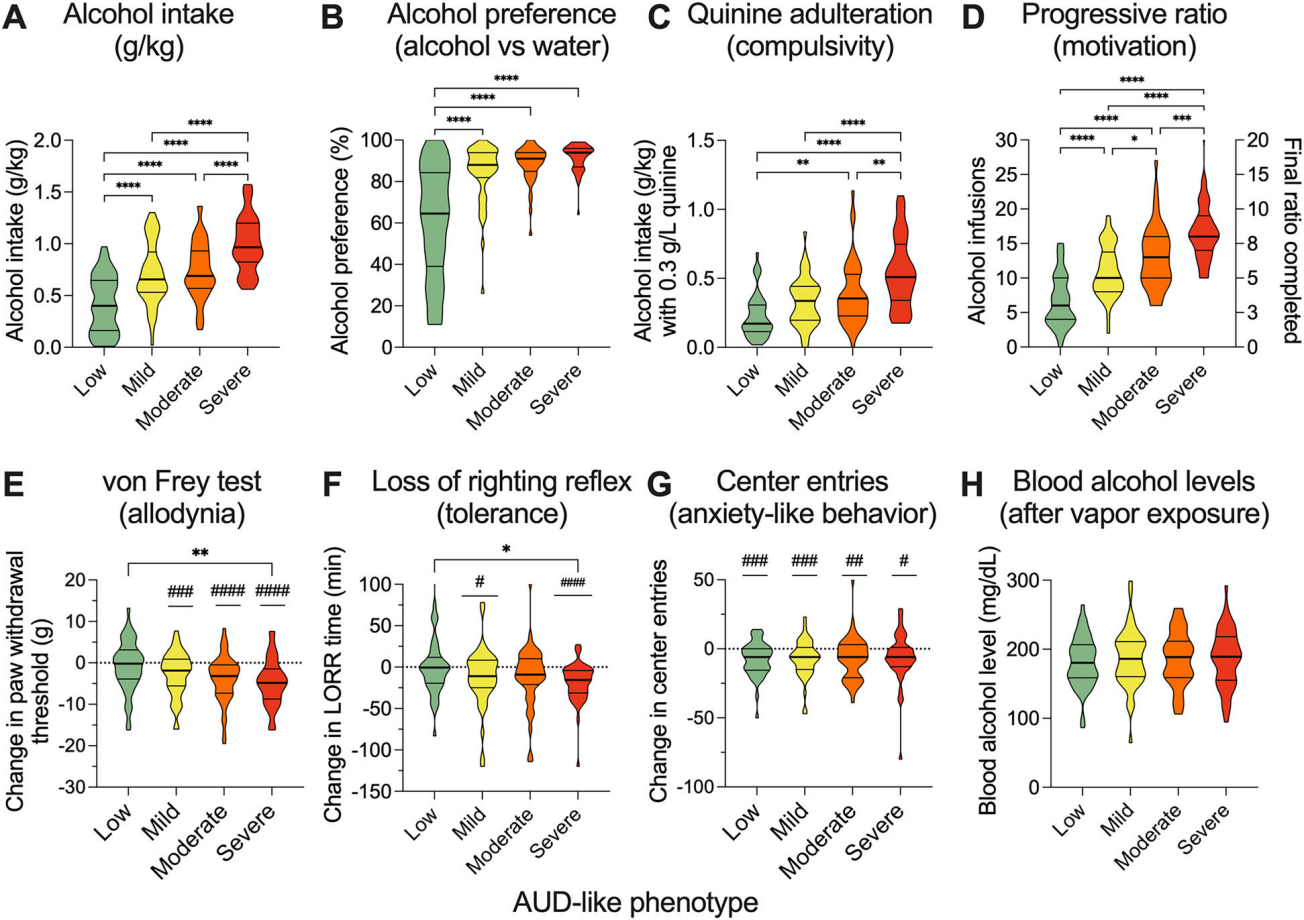
Comparisons between (***A***) alcohol intake, (***B***) alcohol preference, (***C***) alcohol intake when 0.3 g/L quinine was added to the alcohol, and (***D***) motivation to obtain alcohol using the progressive ratio schedule of reinforcement during dependence period in rats characterized as having a low (green), mild (yellow), moderate (orange), or severe (red) AUD-like phenotype. Comparisons in (***E***) change in paw withdrawal threshold after dependence as a measure of withdrawal-induced allodynia, (***F***) change in loss of righting reflex duration as a measure of tolerance, and (***G***) change in center entries in an open field as a measure of anxiety-like behavior in rats with a low (green), mild (yellow), moderate (orange), or severe (red) AUD-like phenotype. ***H***, Blood alcohol levels at the end of the 14 h alcohol vapor exposure in rats within rats with a low (green), mild (yellow), moderate (orange), or severe (red) AUD-like phenotype. In violin plots, thick lines indicate median and thin lines indicate quartiles. Data represent combined results 190 rats, with equal numbers of males (*n* = 95) and females (*n* = 95). Each group (low, mild, moderate, severe) represent *n* = 42–48 rats, depending on exclusions. *p < 0.05; **p < 0.01; ***p < 0.001; ****p < 0.0001 for Sidak's multiple-comparison tests. ^#^*p* < 0.05; ^##^*p* < 0.01; ^###^*p* < 0.001; ^####^*p* < 0.0001 versus 0 for one-sample *t* test.

**Figure 4.**
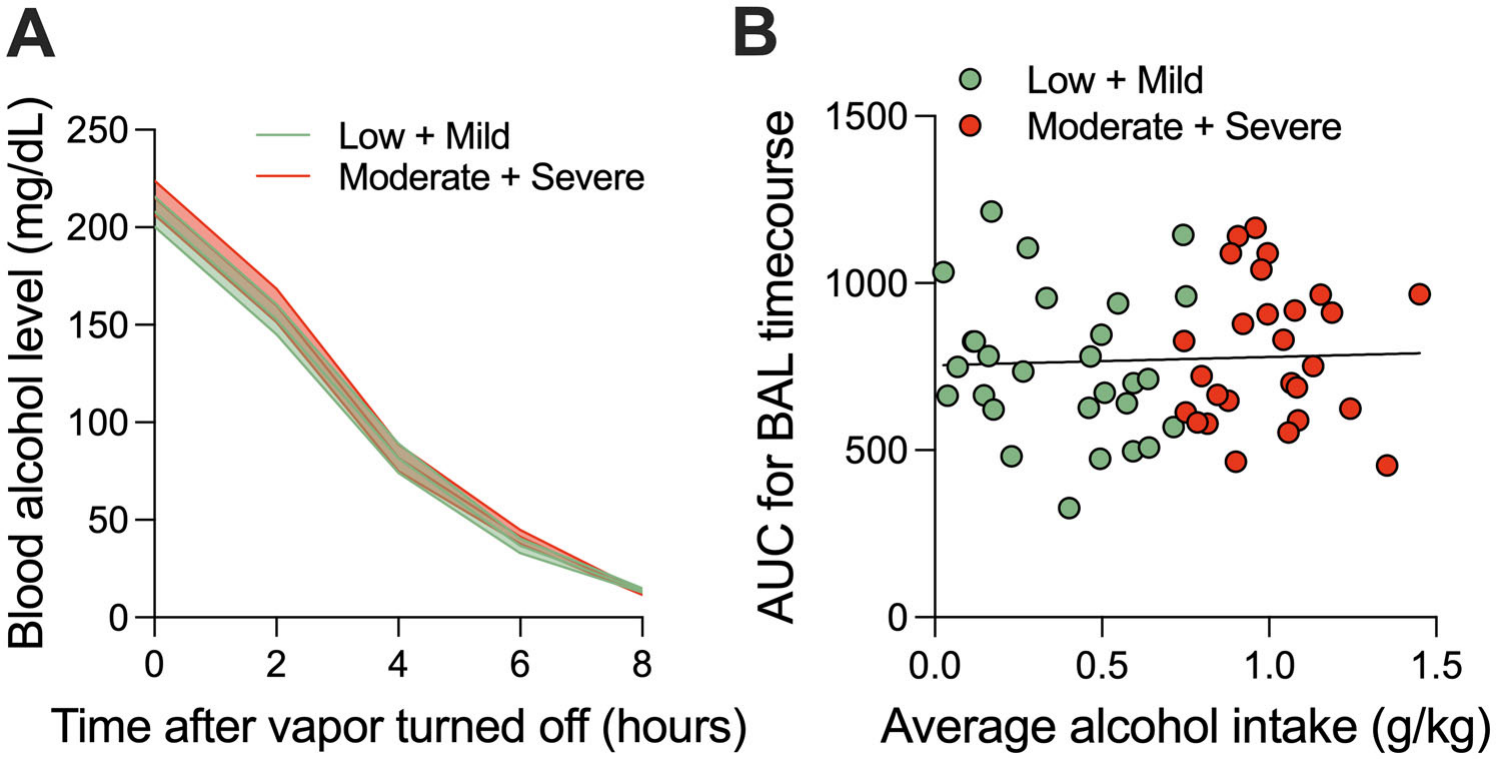
***A***, Time course of blood alcohol level (BAL) decay after the end of the alcohol vapor in rats that had a low or mild phenotype (green) versus moderate or severe phenotype (red; *n* = 56; 28 males, 28 females, from one cohort). ***B***, Lack of relationship between average alcohol intake during dependent self-administration and the AUC of the area under the curve (AUC) for the BAL time course (*n* = 56; 28 males, 28 females, from one cohort).

For data in Figure 2, pre- and post-dependence periods included sessions 7–9 and 10–12, respectively, immediately before the progressive ratio test. The study used two cohorts of 96 heterogeneous stock (HS) rats (48 males, 48 females each), totaling 192 rats. After two rats were euthanized during chronic intermittent ethanol vapor exposure and minor exclusions, data from 190 rats (95 males, 95 females) were analyzed for Figures 2 and 3, with sample sizes detailed in figure legends. For Figure 4, 56 rats (28 males, 28 females) from one cohort were used for the alcohol metabolism study.

Data exclusions due to technical issues or insufficient collection ensured measurement reliability:

Alcohol Preference: Excluded four rats with ≤3 rewards in the final three sessions.

Loss of Righting Reflex (LORR): Excluded 13 measurements due to improper intraperitoneal injection.

Open Field Test: Excluded seven values due to ANY-maze software tracking failures.

Blood Alcohol Levels (BALs): Excluded two measurements due to insufficient blood or assay failure.

Exclusions were <5% of total data points per test, ensuring reliable results.

For Figure 2, pre- and post-dependence data were analyzed using a paired *t* test. For Figure 3, AUD-like phenotype data were analyzed with one-way ANOVA followed by Sidak's multiple-comparison tests. Changes from baseline (Fig. 3*E–G*) were assessed with a one-sample *t* test against 0. For Figure 4, BAL time course data were analyzed using mixed-effects analysis. Area under the curve was calculated per rat, plotted against 3 d average post-dependence intake, and evaluated with linear regression.

AUD-like phenotype severity was determined by *z*-scoring six measures: average alcohol intake (sessions 10–12), progressive ratio reinforcers, alcohol consumption with 0.3 g/L quinine, intake escalation from pre- to post-dependence, LORR tolerance, and von Frey allodynia. *Z*-scores were computed within sex and cohort to address sex differences and cohort effects. The average of these measures formed an overall addiction index (Kallupi et al., 2020; de Guglielmo et al., 2024). The assignment to low, mild, moderate, or severe was based on dividing each cohort and sex into quartiles where the quartile with the lowest value was named low and the highest was named severe. All statistical analyses were performed using GraphPad Prism 10 (GraphPad Software). Statistical significance was set at *p* < 0.05.

## Results

### Characterization of alcohol addiction-like behaviors

The AUD behavioral characterization timeline is shown in Figure 1. Annually, three cohorts of 96 HS rats (48 males, 48 females) are phenotyped. Von Frey (mechanical sensitivity) and open field (anxiety-like behavior) tests occur at baseline (pre-ethanol self-administration) and during acute withdrawal (7–10 h post-ethanol vapor). Rats are trained for 1 week to self-administer oral solutions (0.1 ml of 10% v/v ethanol or tap water), followed by a 3 week self-administration protocol. Loss of righting reflex (LORR) is tested post-pre-dependence drinking to avoid aversive ethanol exposure before drinking behavior. Over 2 weeks, ethanol vapor concentrations are increased, with rats exposed for 14 h/day thereafter. Behavioral tests (self-administration, open field, von Frey) occur during acute withdrawal. Post-self-administration, a second LORR test assesses tolerance. Rats are euthanized during intoxication (13–15 h of vapor exposure), acute withdrawal (7–10 h post-vapor), or protracted abstinence (4 weeks post-vapor).

Data presented in Figures 2–4 are derived from two cohorts of HS rats (*n* = 190), providing a robust initial dataset from the Alcohol Biobank, which continues to expand with additional cohorts.

### Effects pre- and post-dependence using CIE

HS rats (*n* = 190) went through the procedure as described and measured obtained during the pre- and post-dependence phases were evaluated (Fig. 2). Rats self-administered more ethanol (*t*_(189)_ = 8.280; *p* < 0.0001; Fig. 2*A*), had greater preference for ethanol (*t*_(185)_ = 6.881; *p* < 0.0001; Fig. 2*B*), consume more ethanol when quinine is added (*t*_(189)_ = 3.530; *p* = 0.0005; Fig. 2*C*), and showed more motivation to obtain ethanol under the progressive ratio schedule of reinforcement (*t*_(189)_ = 8.013; *p* < 0.0001; Fig. 2*D*) after induction of dependence compared with their own pre-dependence baseline.

Paw withdrawal threshold in the von Frey test (*t*_(189)_ = 7.161; *p* < 0.0001; Fig. 2*E*), time of loss of righting reflex as a measure of alcohol sensitivity (*t*_(176)_ = 4.008; *p* < 0.0001; Fig. 2*F*), and entries into the center of an open field as a measure of anxiety-like behavior (*t*_(182)_ = 6.218; *p* < 0.0001; Fig. 2*G*) all decreased after induction of dependence compared with the pre-dependence baseline. Blood alcohol levels were measured after alcohol dependence and were, on average, 184.7 ± 2.83 (Fig. 2*H*).

### Individual differences in phenotype severity

We calculated an “addiction index” for each of the rats and separated them into quartiles classified as low, mild, moderate, or severe, where rats with the lowest addiction index were considered low and those with the highest were severe. As expected, there were substantial individual differences and the groups differed significantly across all self-administration procedures, including alcohol intake (*F*_(3,187)_ = 35.56; *p* < 0.0001; Fig. 3*A*), alcohol preference (*F*_(3,186)_ = 37.14; *p* < 0.0001; Fig. 3*B*), alcohol consumption when adulterated with quinine (*F*_(3,186)_ = 19.14; *p* < 0.0001; Fig. 3*C*), and responding under a progressive ratio schedule (*F*_(3,186)_ = 50.71; *p* < 0.0001; Fig. 3*D*).

There was also a significant effect of group when evaluating withdrawal-induced allodynia (*F*_(3,185)_ = 4.208, *p* = 0.0066; Fig. 3*E*). All groups also showed significant allodynia (mild: *t*_(47)_ = 3.511, *p* = 0.001; moderate: *t*_(46)_ = 4.677, *p* < 0.0001; severe: *t*_(45)_ = 5.681; *p* < 0.0001)), except the low group (*t*_(47)_ = 0.8823, *p* = 0.3821; Fig. 3*E*). For the tolerance measure, there was a significant effect of group (*F*_(3,143)_ = 4.404, *p* = 0.0054; Fig. 3*F*) where the severe group had greater tolerance than the low group (*p* = 0.0346). There was significant tolerance observed in the mild and severe groups (mild: *t*_(44)_ = 2.233, *p* = 0.0307; severe: *t*_(45)_ = 5.314; *p* < 0.0001; Fig. 3*F*). Tolerance trended toward significance in the moderate group (*t*_(42)_ = 1.936, *p* = 0.0596), but there was no observed tolerance in the low group (*t*_(43)_ = 0.093, *p* = 0.927; Fig. 3*F*). There was no effect of group when evaluating center entries in an open field test as a measure of anxiety-like behavior (*F*_(3,178)_ = 0.08682; *p* = 0.9672; Fig. 3*G*); however, all groups showed significant decreases in center entries (low: *t*_(44)_ = 3.983, *p* = 0.0003; mild: *t*_(46)_ = 3.688, *p* = 0.0006; moderate: *t*_(44)_ = 2.890, *p* = 0.0060; severe: *t*_(44)_ = 2.251, *p* = 0.0295). The behavioral differences between the groups were not mediated by group differences in blood alcohol levels generated by the CIE model (*F*_(3,187)_ = 0.2109; *p* = 0.8887; Fig. 3*H*).

### Blood alcohol level time course and relationship to self-administration

To confirm rats were in withdrawal during the behavioral experiments (conducted 7–10 h after vapor turned off) and evaluate the potential effects of ethanol metabolism on the behavioral assays, blood alcohol levels (BALs) were measured in a subset of rats (*n* = 56) every 2 h beginning from when the ethanol vapor ended until 8 h later. There was a main effect of time (*F*_(4,210)_ = 585.0; *p* < 0.0001), but no effect of phenotype or interaction (Fig. 4*A*). There was no significant relationship between average ethanol intake in the dependence period and BAL time course AUC (*R*^2^ = 0.00019; *p* = 0.752; Fig. 4*B*).

## Discussion

The Alcohol Biobank constitutes a pivotal resource for advancing AUD research, complementing established cocaine and oxycodone biobanks (Carrette et al., 2021). Over the past 5 years, these repositories have distributed over 2,000 samples to 40 investigators across the United States and Europe, catalyzing significant advances in addiction neuroscience (Carrette et al., 2022; Duttke et al., 2022; Kumaresan et al., 2023; Zhou et al., 2023; Okamoto et al., 2024; Vu et al., 2025). The Alcohol Biobank extends this paradigm, providing thousands of samples from over 700 genetically diverse HS rats, with data from two cohorts (*n* = 190) reported herein. By facilitating comparative analyses across substances, the Alcohol Biobank enables elucidation of shared and distinct neurobiological mechanisms underlying alcohol, cocaine, and oxycodone addiction. Samples are characterized with phenotypic and genotypic data, including metadata (animal ID, sex, age, body weight, sample weight, collection date, preservation timestamps). RFID chips and barcodes ensure traceability throughout the sample lifecycle.

Longitudinal samples and terminal samples are collected at critical experimental time points: baseline, pre-dependence, dependence, intoxication, acute withdrawal, protracted abstinence, or from naive controls. Preservation via snap-freezing or paraformaldehyde fixation supports diverse applications, including genomics, transcriptomics, proteomics, metabolomics, and histological studies. The CIE model, a validated standard in AUD research (Roberts et al., 2000; Gilpin et al., 2008; Vendruscolo and Roberts, 2014; de Guglielmo et al., 2016, 2019, 2023; Doyle et al., 2024), underpins the Alcohol Biobank's behavioral phenotyping, capturing escalation of ethanol consumption, compulsive drinking, and withdrawal symptoms such as allodynia, anxiety-like behavior, and tolerance. This multifaceted approach aligns with at least five DSM-5 criteria for AUD, enhancing translational relevance (Nieto et al., 2021). Moreover, the stratification into low, mild, moderate, and severe quartiles based on the addiction index allows for the modeling of varying degrees of AUD severity, with the severe group exhibiting more pronounced AUD-like endophenotypes analogous to severe AUD in humans meeting multiple DSM-5 criteria. The use of *z*-scores within each cohort and sex for the addiction index ensures reliable quartile assignments across cohorts, as evidenced by consistent behavioral differences in combined data (Fig. 3).

Data from two cohorts (*n* = 190) provide robust insights into AUD-like behaviors. Post-dependence, rats exhibited significant escalation in ethanol intake, heightened preference, sustained consumption despite quinine adulteration, and increased motivation under progressive ratio schedules (Fig. 2*A–D*), indicative of compulsive drinking and tolerance, hallmark features of AUD (Koob and Volkow, 2010). Withdrawal-induced manifestations, including mechanical allodynia, reduced loss of righting reflex duration, and diminished open-field center entries (Fig. 2*E–G*), underscore the negative affective and somatic states associated with dependence (Koob, 2022). These findings, enabled by the Alcohol Biobank's standardized CIE protocol and comprehensive phenotyping, establish a robust platform for identifying biomarkers of compulsive drinking and withdrawal severity.

Individual differences in AUD-like phenotype severity (Fig. 3) further highlight the Alcohol Biobank's utility. Rats classified as moderate or severe exhibited significantly elevated ethanol intake, preference, and compulsive drinking compared with low or mild groups (Fig. 3*A–D*), with corresponding increases in allodynia and tolerance (Fig. 3*E*,*F*). Notably, anxiety-like behavior was consistent across groups (Fig. 3*G*), suggesting a pervasive withdrawal effect amenable to molecular and neural dissection using Alcohol Biobank samples. The absence of a correlation between BECs and ethanol intake (Fig. 4*B*) indicates that behavioral variability is driven by pharmacodynamic and genetic factors rather than pharmacokinetic differences, facilitating targeted investigations using Alcohol Biobank resources. Differences observed among rats partially reflect the genetic diversity among HS rats and supports ongoing studies aimed at identifying specific alleles that underlie behavioral and physiological differences.

The Alcohol Biobank's samples are currently supporting cutting-edge investigations, as summarized in Table 2, including microbiome profiling to elucidate gut–brain interactions, metabolomics to identify AUD-related metabolic signatures, proteomics to uncover protein dysregulation, single-cell RNA sequencing to map cellular heterogeneity in brain regions such as the amygdala and nucleus accumbens, neuroanatomical studies to delineate circuit alterations, in vivo MRI to assess structural and functional brain changes, whole-brain imaging to visualize neural adaptations, and RNA methylation to investigate epigenetic modifications. These applications address critical questions regarding AUD susceptibility, progression, and therapeutic response, with potential to identify novel targets for mitigating compulsive drinking or withdrawal-induced allodynia.

**Table 2.** Ongoing collaborations utilizing Alcohol Biobank samples in AUD research

Sample type	Preservation technique	Research application
Feces and caecum	Snap-frozen	Microbiome profiling: Studying gut–brain interactions in AUD-related behaviors
Blood (plasma, serum)	Snap-frozen	Metabolomics: Identifying AUD-related metabolic signatures and biomarkers
Brain (various regions)	Snap-frozen	Proteomics: Uncovering protein dysregulation in AUD pathways
Brain (amygdala, nucleus accumbens)	Snap-frozen	Single-cell RNA sequencing: Mapping cellular heterogeneity in AUD brain regions
Brain (various regions)	Paraformaldehyde fixation	Neuroanatomical studies: Delineating circuit alterations in AUD
Live animals	None	In vivo MRI: Assessing structural and functional brain changes in AUD
Brain (whole)	Paraformaldehyde fixation	Whole-brain imaging: Visualizing neural adaptations in AUD
Brain (amygdala)	Snap-frozen	RNA methylation: Investigating epigenetic modifications in AUD-related brain regions

Human biobanks, such as the NIAAA's COGA (Begleiter, 1995), provide genotypic and phenotypic data for AUD research, with COGA including interviews and electrophysiological measures from 17,000 individuals (Dick et al., 2023). However, human biobank sample acquisition faces significant barriers (Coppola et al., 2019; Annaratone et al., 2021; Nieto et al., 2021). Ethical and legal requirements, including informed consent, IRB approvals, GDPR compliance, and pseudonymization, create complex governance and access delays (Harris et al., 2012; Coppola et al., 2019; Chandrashekar et al., 2022). Heterogeneous protocols hinder standardization, limiting data comparability (Coppola et al., 2019). Pre-analytical variability, such as postmortem delays, degrades sample quality, impeding molecular analyses (Shabihkhani et al., 2014; Coppola et al., 2019; Annaratone et al., 2021). High costs and underuse, as seen in the EFS Centre-Atlantique biobank, challenge sustainability (Sapey et al., 2016; Annaratone et al., 2021). Self-reported data, which may be prone to bias, reduce AUD phenotyping precision (Greenfield and Kerr, 2008; Nieto et al., 2021). Conversely, the preclinical Alcohol Biobank's standardized CIE model, immediate sample collection, cost-effective open-access framework, and detailed phenotyping, including brain tissue access, provide high-fidelity data for AUD research. By integrating findings from preclinical and human biobanks, researchers can bridge translational gaps, leveraging the controlled, high-fidelity data from the Alcohol Biobank to complement the broader but less precise datasets from human repositories, thereby advancing the understanding and treatment of AUD.

Our behavioral protocol while robust has its own limitations. The CIE model do not fully recapitulate the human condition (Nieto et al., 2021) and involves forced exposure to high concentrations of ethanol vapor over protracted periods. This obligate exposure may introduce additional stress compared with voluntary drinking models. Despite this, the CIE model is a well-validated standard in AUD research, reliably producing escalated ethanol consumption and withdrawal symptoms that align with DSM-5 criteria (Rodd et al., 2005). The HS rat model, despite genetic diversity, may not replicate human AUD's sociocultural dimensions, requiring integration with human studies for translational relevance. Controlled experimental conditions, while minimizing variability, cannot fully account for complex environmental factors influencing human AUD (Koob and Volkow, 2010). These constraints highlight the need for complementary human research to validate preclinical findings.

The Alcohol Biobank project aims to collect and phenotype samples from 1,000 rats, with all samples stored indefinitely to support long-term research. The possibility of expanding the collection beyond 1,000 rats will depend on securing additional funding to sustain and grow the biobank, which we anticipate will be influenced by the interest and utilization of the biobank by the research community.

In summary, the Alcohol Biobank enhances AUD research by providing samples from genetically diverse rats. Its open-access model promotes global collaboration (Carrette et al., 2021). With rigorous behavioral phenotyping and sample preservation, it supports training researchers and drives progress in AUD understanding and management.

## Synthesis

Reviewing Editor: Laura Bradfield, University of Technology Sydney Decisions are customarily a result of the Reviewing Editor and the peer reviewers coming together and discussing their recommendations until a consensus is reached. When revisions are invited, a fact-based synthesis statement explaining their decision and outlining what is needed to prepare a revision will be listed below. The following reviewer(s) agreed to reveal their identity: Andrew Lawrence.

Thank you for your manuscript regarding the establishment of an Alcohol Biobank with samples from over 700 genetically diverse heterogeneous stock rats. The reviewers and I agree that this is a high quality manuscript reporting an important resource that will advance preclinical research into alcohol use disorder.

The reviewers raised only minor comments, which you can read in their detailed reviews below. However, upon consultation, the reviewers and I identified a few additional points requiring clarification, particularly regarding sample sizes. Although it is stated that 190 rats contributed to the data, it is unclear to us which specific cohorts were used for each dataset and figure. We request that the sample sizes are included for each graph in the Figure legends, including numbers of male and female rats.

Relatedly, reviewer #2 asks about longevity of the Biobank. For clarification, this question refers to whether this is an ongoing project, and if so, how long you intend on running cohorts for. Regardless of whether the project has a definite or indefinite timeline, we request that this be stated more clearly in the manuscript.

Thank you for the interesting manuscript and contributing this important resource to the field.

Reviewer #1

This is an interesting manuscript that describes a much-needed resource for the preclinical alcohol research. I commend the authors for this endeavour and have only a few minor comments that should be addressed prior to publication.

1. The tense switches from past to present in various places, please amend for consistency.

2. For the open field test please report the luminosity (lux) for the room lighting at time of test.

3. For self-administration after abstinence, was this also re-assessed following protracted abstinence? This would be most useful but it is not clear - at present it reads as though this was only re-assessed after acute withdrawal. If not tested to date will the authors add this additional group?

4. For the section "Euthanasia during intoxication, acute withdrawal, or protracted abstinence" please clarify that presumably these rats did not undergo any self-administration sessions after induction of dependence.

5. For the 4% PFA please indicate what buffer (PBS?) &pH the solution was in.

6. For all various exclusion criteria (preference measure, LORR, maze software etc) please explain the issues more clearly and indicate how many were excluded.

7. Likewise for missing BAL measures.

8. The assignment of "AUD severity" the authors divided cohorts into quartiles. How reliable was this between cohorts in terms of behavioral traits and how do the 4 quartiles relate to DSM-5 in terms of endophenotype displayed?

9. It would be useful for the statistical software package used to be named.

Reviewer #2

Establishment of a biobank seems like an important tool for collaboration and advancement of preclinical research into alcohol use disorder. On the whole, I thought that the manuscript was well-written, and clearly show the importance of such a tool as well as outlining how the intial data set was established.

I was a little unclear about the dependence phase. During this phase it's stated that rats are housed in vapour chambers - is this for the whole time throughout this period, or just for the 14 hours of vapour exposure? Are they still pair-housed while in the vapour chambers? If not, could the stress of social isolation impact the biomarkers in samples taken?

Further, although I realise this is a well-established model of alcohol dependency, I think that the potential stress invoked by obligate intoxication through exposure to eventual high concentrations of ethanol vapour over protracted periods is worth discussing along with other limitations.

I would also be interested in the longevity of this project, although I realise that this may depend somewhat on how much interest it generates going forward.

## Author Response

Response to Reviewers We sincerely thank the Editor and the Reviewers for their constructive and insightful feedback on our manuscript, "A Preclinical Alcohol BioBank: Samples from Behaviorally Characterized HS Rats for AUD Research". These comments have significantly improved the clarity, rigor, and transparency of our work, ensuring its alignment with eNeuro's standards for innovative neuroscience research. We have carefully addressed all reviewer comments, incorporating revisions marked in blue text within the manuscript to highlight changes. Below, we provide a detailed point-by-point response to each comment, outlining how we have clarified methodological details, enhanced discussions, and ensured consistency to strengthen the manuscript's contribution to addiction biology.

Synthesis of Reviews:

Significance Statement Comments for Author (Required):

The significance statement is good, but there is a typo in the word 'advance'. No other changes are needed.

Response: We thank the editor for pointing out this typo and we have fixed it accordingly.

Comments on the Visual Abstract for Author (Required):

N/A Synthesis Statement for Author (Required):

Thank you for your manuscript regarding the establishment of an Alcohol Biobank with samples from over 700 genetically diverse heterogeneous stock rats. The reviewers and I agree that this is a high-quality manuscript reporting an important resource that will advance preclinical research into alcohol use disorder.

Response: We thank the Editor and the Reviewers for the positive evaluation of the manuscript.

The reviewers raised only minor comments, which you can read in their detailed reviews below.

However, upon consultation, the reviewers and I identified a few additional points requiring clarification, particularly regarding sample sizes. Although it is stated that 190 rats contributed to the data, it is unclear to us which specific cohorts were used for each dataset and figure. We request that the sample sizes are included for each graph in the Figure legends, including numbers of male and female rats.

Response: We thank the Editor and the Reviewers for their suggestion to clarify the sample sizes and cohort composition for each dataset and figure in the Alcohol Biobank study. To address this, we have updated the figure legends for Figures 2, 3, and 4 to include specific sample sizes and the number of male and female rats for each panel. The study utilized two cohorts, each consisting of 96 heterogeneous stock (HS) rats (48 males and 48 females per cohort), totaling 192 rats initially. Due to two rat's death during the chronic intermittent ethanol (CIE) vapor 2 exposure phase and other minor exclusions, data from 190 rats (95 males and 95 females) were included in the analyses for Figures 2 and 3, which present combined results from both cohorts.

Specific sample sizes for each panel vary slightly due to test-specific exclusions, as detailed in the "Statistical analysis" subsection of the Methods section. For Figure 4, which presents the alcohol metabolism study, a subset of 56 rats (28 males and 28 females) from one of the two cohorts was used, as specified in the legend.

Reviewer #2 also asked about the longevity of the Biobank. For clarification, this question refers to whether this is an ongoing project, and if so, how long you intend on running cohorts for.

Regardless of whether the project has a definite or indefinite timeline, we request that this be stated more clearly in the manuscript.

Response: This point has been addressed in the specific comment from reviewer 2 (see below) and more details have been added to the discussion.

Reviewer #1 This is an interesting manuscript that describes a much-needed resource for the preclinical alcohol research. I commend the authors for this endeavour and have only a few minor comments that should be addressed prior to publication.

Response: We thank the reviewer for the positive evaluation of the manuscript.

1. The tense switches from past to present in various places, please amend for consistency.

Response: We thank the reviewer for highlighting the issue of tense inconsistencies in the manuscript. To address this, we have carefully reviewed the manuscript and revised sections where present tense was inadvertently used to describe study-specific actions, particularly in the Methods and Results sections. These sections now consistently use past tense to describe procedures and findings specific to this study. Present tense has been retained for general descriptions of the heterogeneous stock (HS) rat model and standard procedures to clearly distinguish between general practices and study-specific actions 2. For the open field test please report the luminosity (lux) for the room lighting at time of test.

Response: We thank the reviewer for this question. The luminosity (lux) for the room lighting at time of test was measured through a Digital Lux Meter (Dr. Meter, model LX1330B) and set at 20 Lux. This information has been added to the methods.

3. For self-administration after abstinence, was this also re-assessed following protracted abstinence? This would be most useful but it is not clear - at present it reads as though this was only re-assessed after acute withdrawal. If not tested to date will the authors add this additional group? 3 Response: We thank the reviewer for seeking clarification on whether self-administration was reassessed following protracted abstinence. We confirm that self-administration was not reassessed during the 4-week protracted abstinence period, and this was an intentional design choice. Our goal was to provide biobank users with samples from rats in a true state of protracted abstinence, free from the confounding effects of recent alcohol exposure. This approach ensures that researchers can investigate molecular and neurobiological changes associated with prolonged abstinence without the influence of additional ethanol self-administration, which is critical for studies exploring recovery mechanisms or therapeutic interventions for alcohol use disorder. To clarify this in the manuscript, we have added a sentence to the "Self-administration during ethanol dependence" subsection of the Methods section, stating: "No self-administration sessions were conducted during the 4-week protracted abstinence period for rats assigned to this group, ensuring samples reflect a state of prolonged abstinence without recent ethanol exposure." 4. For the section "Euthanasia during intoxication, acute withdrawal, or protracted abstinence" please clarify that presumably these rats did not undergo any self-administration sessions after induction of dependence.

Response: We appreciate the reviewer's request for clarification regarding self-administration sessions after the induction of dependence in the “Euthanasia during intoxication, acute withdrawal, or protracted abstinence” section. We wish to clarify that all rats, including those euthanized during intoxication, acute withdrawal, or protracted abstinence, underwent self-administration sessions after induction of dependence, specifically during the dependence phase.

As described in the "Self-administration during ethanol dependence" subsection, rats completed 17 self-administration sessions over approximately 3 weeks during acute withdrawal periods (7- 10 hours post-vapor) following chronic intermittent ethanol vapor exposure (CIE). For rats in the protracted abstinence group, these self-administration sessions occurred before the 4-week abstinence period, during which no further ethanol exposure or self-administration took place.

The reviewer may have interpreted the euthanasia timing as implying no self-administration occurred post-dependence, particularly for the protracted abstinence group. To address this, we have revised the "Euthanasia during intoxication, acute withdrawal, or protracted abstinence" subsection to include: "All rats underwent self-administration sessions during the dependence phase post-CIE induction, as described in the 'Self-administration during ethanol dependence' subsection. For rats euthanized during protracted abstinence, no self-administration or ethanol exposure occurred during the 4-week abstinence period prior to euthanasia." This revision clarifies the timeline and ensures alignment with the experimental design.

5. For the 4% PFA please indicate what buffer (PBS?) &pH the solution was in.

Response: The 4% PFA solution was prepared in phosphate-buffered saline (PBS) at pH 7.4. To address this in the manuscript, we have revised the "Euthanasia during intoxication, acute withdrawal, or protracted abstinence" section in the Methods to explicitly state: "For fixed samples, rats were euthanized using CO2 exposure and then intracardially perfused with 150 ml of ice-cold saline followed by 350 ml of 4% paraformaldehyde in phosphate-buffered saline (PBS, pH 7.4) before tissue was collected. Fixed brains were post-fixed in 4% PFA in PBS at 4{degree sign}C for 24-72 hours before cryoprotection in 30% sucrose with 0.1% sodium azide in PBS".

4 6. For all various exclusion criteria (preference measure, LORR, maze software etc) please explain the issues more clearly and indicate how many were excluded.

Response: We appreciate the reviewer's request for clearer explanations of the exclusion criteria and the number of excluded data points for behavioral tests in the Alcohol Biobank study. To address this, we have revised the "Statistical analysis" subsection in the Methods section to include a detailed paragraph outlining the specific reasons for exclusions and the number of excluded data points for each test across the two cohorts (n=190 rats). Specifically, two animals were excluded from the study due to death during chronic intermittent ethanol vapor exposure (CIE). Additionally, for specific tests, we excluded: 4 alcohol preference values (rats received {less than or equal to}3 rewards in each of the final three sessions), 13 Loss of Righting Reflex (LORR) measurements (issues with intraperitoneal injection), 7 open field test values (ANY-maze software tracking failures due to camera issues), and 2 blood alcohol level (BAL) measurements (insufficient blood collected). These exclusions represent less than 5% of the total data points for each test, ensuring that the reported results are based on reliable, high-quality measurements. This low exclusion rate is typical in behavioral pharmacology studies and does not bias the findings.

7. Likewise for missing BAL measures.

Response: As detailed in the revised manuscript and in the previous answer, two BAL measurements were excluded due to technical issues with the Analox Alcohol Analyser or insufficient blood collection.

8. The assignment of "AUD severity" the authors divided cohorts into quartiles. How reliable was this between cohorts in terms of behavioral traits and how do the 4 quartiles relate to DSM-5 in terms of endophenotype displayed? Response: We thank the reviewer for their insightful questions regarding the reliability of the AUD severity assignment across cohorts and the relation of the four quartiles to DSM-5 criteria in terms of endophenotypes displayed in the Alcohol Biobank study.

Regarding the reliability of the AUD severity assignment across cohorts, the addiction index was calculated by averaging z-scores of six behavioral measures (average alcohol intake, progressive ratio responding, quinine-adulterated consumption, escalation of intake, tolerance in the loss of righting reflex task, and allodynia in the von Frey test) within each cohort and sex. This standardization accounts for potential cohort effects and sex differences, ensuring that the quartiles (low, mild, moderate, severe) represent consistent relative severities within each cohort.

When data from the two cohorts (n=190) were combined, as shown in Figure 3, the behavioral traits exhibited clear and significant differences across the quartiles, indicating that the classification is robust and consistent across cohorts. To further confirm reliability, cohort-specific analyses (not shown) revealed comparable mean values for key behavioral measures (e.g., ethanol intake, preference) across cohorts for each quartile, supporting the consistency of the severity assignments.

5 Concerning the relation of the four quartiles to DSM-5 criteria in terms of endophenotypes displayed, our behavioral assessments were designed to model key aspects of AUD as defined by the DSM-5. The measures included in the addiction index correspond to eight DSM-5 criteria, such as drinking more or longer than intended (escalated intake), continued use despite negative consequences (quinine-adulterated consumption), tolerance (loss of righting reflex), and withdrawal symptoms (mechanical allodynia, anxiety-like behavior). The stratification into low, mild, moderate, and severe quartiles reflects a spectrum of AUD-like endophenotypes, with the severe quartile exhibiting more pronounced behaviors, such as significantly higher ethanol intake, preference, and withdrawal symptoms (Fig. 3A-F), analogous to individuals with severe AUD who meet six or more DSM-5 criteria. While direct mapping to human diagnostic criteria is not feasible due to differences between rodent and human behaviors, this approach provides a translational framework for studying the neurobiological underpinnings of AUD severity, facilitating multi-omics analyses to identify novel therapeutic targets. To clarify this in the manuscript, we have added a statement to the Discussion section: "Moreover, the stratification into low, mild, moderate, and severe quartiles based on the addiction index allows for the modeling of varying degrees of AUD severity, with the severe group exhibiting more pronounced AUD-like endophenotypes, such as escalated ethanol intake, compulsive drinking despite quinine adulteration, and withdrawal symptoms, analogous to severe AUD in humans meeting multiple DSM-5 criteria. The use of z-scores within each cohort and sex for the addiction index ensures reliable quartile assignments across cohorts, as evidenced by consistent behavioral differences in combined data." 9. It would be useful for the statistical software package used to be named.

Response: We thank the reviewer for noticing this missing information. All statistical analyses were performed using GraphPad Prism 10 (GraphPad Software, San Diego, CA). This info has been added to the statistical analysis section.

Reviewer #2 Establishment of a biobank seems like an important tool for collaboration and advancement of preclinical research into alcohol use disorder. On the whole, I thought that the manuscript was well-written, and clearly show the importance of such a tool as well as outlining how the intial data set was established.

Response: We thank the reviewer for the positive evaluation of the manuscript I was a little unclear about the dependence phase. During this phase it's stated that rats are housed in vapor chambers - is this for the whole time throughout this period, or just for the 14 hours of vapor exposure? Are they still pair-housed while in the vapour chambers? If not, could the stress of social isolation impact the biomarkers in samples taken? Response: We appreciate the Reviewer's request for clarification regarding the housing conditions during the dependence phase of the Alcohol Biobank study. To address the reviewer's questions, we confirm that rats were pair-housed (two per cage) in ethanol vapor chambers (La 6 Jolla Alcohol Research Inc, La Jolla, CA) throughout the entire dependence phase. Ethanol vapor was circulated for 14 hours/day, with a 10-hour off period each day, during which the rats remained in the chambers. Rats were only removed from the chambers for self-administration sessions, which occurred during acute withdrawal (7-10 hours after vapor exposure). This continuous housing in the vapor chambers, with pair-housing maintained throughout, minimizes handling stress and ensures consistent exposure conditions, which is standard practice in the chronic intermittent ethanol (CIE) vapor exposure model. We have edited the methods to reflect this.

Further, although I realize this is a well-established model of alcohol dependency, I think that the potential stress invoked by obligate intoxication through exposure to eventual high concentrations of ethanol vapor over protracted periods is worth discussing along with other limitations.

Response: We thank the reviewer for this point. The discussion has been updated to reflect this comment. Now it reads: "Additionally, the CIE model involves forced exposure to high concentrations of ethanol vapor over protracted periods, inducing cycles of intoxication and withdrawal designed to replicate the stress and negative affect experienced in human AUD. This obligate exposure may introduce additional stress compared to voluntary drinking models.

Despite this, the CIE model is a well-validated standard in AUD research, reliably producing escalated ethanol consumption and withdrawal symptoms that align with DSM-5 criteria (Rodd et al., 2005)" I would also be interested in the longevity of this project, although I realize that this may depend somewhat on how much interest it generates going forward.

Response: We appreciate the reviewer's interest in the longevity of the Alcohol Biobank. The project is committed to collecting and phenotyping samples from 1000 heterogeneous stock (HS) rats, with all samples stored indefinitely in the biobank to ensure long-term availability for researchers. This target builds on the current collection of over 700 rats, as described in the manuscript, and reflects our goal to provide a robust resource for multi-omics analyses, such as transcriptomics, epigenomics, and metabolomics, to uncover novel therapeutic targets. The possibility of expanding the collection beyond 1000 rats will depend on securing additional funding, which we anticipate will be influenced by the interest and utilization of the biobank by the research community, as evidenced by ongoing collaborations summarized in Table 2. To address this comment, we have added a statement to the Discussion section of the manuscript: "The Alcohol Biobank project aims to collect and phenotype samples from 1000 rats, with all samples stored indefinitely to support long-term research. The possibility of expanding the collection beyond 1000 rats will depend on securing additional funding to sustain and grow the biobank, which we anticipate will be influenced by the interest and utilization of the biobank by the research community".
